# Neutrophil-lymphocyte ratio in metastatic breast cancer is not an independent predictor of survival, but depends on other variables

**DOI:** 10.1038/s41598-019-53606-3

**Published:** 2019-11-18

**Authors:** Alejandra Ivars Rubio, Juan Carlos Yufera, Pilar de la Morena, Ana Fernández Sánchez, Esther Navarro Manzano, Elisa García Garre, Elena García Martinez, Gema Marín Zafra, Manuel Sánchez Cánovas, Esmeralda García Torralba, Francisco Ayala de la Peña

**Affiliations:** 1 Servicio de Hematología y Oncología Médica, Hospital Universitario Morales Meseguer, Murcia, Spain; 2 Instituto Murciano de Investigación Biosanitaria (IMIB-Arrixaca), Murcia, Spain; 3 Universidad de Murcia, Murcia, Spain; 4 Universidad Católica San Antonio de Murcia, Guadalupe, Murcia Spain

**Keywords:** Prognostic markers, Breast cancer

## Abstract

The prognostic impact of neutrophil-lymphocyte ratio (NLR) in metastatic breast cancer (MBC) has been previously evaluated in early and metastatic mixed breast cancer cohorts or without considering other relevant prognostic factors. Our aim was to determine whether NLR prognostic and predictive value in MBC was dependent on other clinical variables. We studied a consecutive retrospective cohort of patients with MBC from a single centre, with any type of first line systemic treatment. The association of NLR at diagnosis of metastasis with progression free survival (PFS) and overall survival (OS) was evaluated using Cox univariate and multivariate proportional hazard models. In the full cohort, that included 263 MBC patients, a higher than the median (>2.32) NLR was significantly associated with OS in the univariate analysis (HR 1.36, 95% CI 1.00–1.83), but the association was non-significant (HR 1.12, 95% CI 0.80–1.56) when other clinical covariates (performance status, stage at diagnosis, CNS involvement, visceral disease and visceral crisis) were included in the multivariate analysis. No significant association was observed for PFS. In conclusion, MBC patients with higher baseline NLR had worse overall survival, but the prognostic impact of NLR is likely derived from its association with other relevant clinical prognostic factors.

## Introduction

Breast cancer is still a major cause of cancer mortality in women. Distant metastases, both at diagnosis and as a result of early breast cancer recurrence, cause most of BC deaths. Although recent therapeutic and diagnostic advances have prolonged survival in metastatic breast cancer (MBC), this is still considered a non-curable disease^1^. Further progress in the treatment of these women will undoubtedly come from a better knowledge of the molecular and biological basis of its clinical behaviour. However, the recent translation of therapeutic advances to the clinical setting with targeted treatments has not been linked to the availability of new biomarkers able to improve prognostic stratification and predict therapeutic response.

The modifications of immune response and inflammation by the tumour are considered hallmarks of cancer^2^, and much work has been developed in this area in recent years, leading to the emergence of immunotherapy as a renewed therapeutic strategy. Chronic inflammation in the tumour microenvironment enhances tumour growth, angiogenesis and tumour cell survival^3^. The contribution of neutrophils to tumour progression has been clearly documented both in the experimental and the clinical setting^4^. Conversely, tumour-infiltrating lymphocytes are paramount players in the immune surveillance of tumours, and the predictive and prognostic impact of lymphocyte infiltration and specific subpopulations has been previously demonstrated in breast cancer^5,6^.

Its easy availability has lead to the evaluation of peripheral blood neutrophil and lymphocyte count as surrogate markers of host inflammation and immune response in cancer patients. Lymphopenia^7,8^ has shown a significant prognostic impact in patients with breast cancer in diverse stages and treatment settings. The combination of both lymphocyte and neutrophil counts in a single measure, the neutrophil-lymphocyte ratio (NLR), is a well established prognostic factor in multiple tumours^9,10^ and in early breast cancer, with higher values of NLR associated to worse outcome, both in Asian^11^ and Western populations^12,13^. A recent metanalysis evaluating NLR in more than 8.000 breast cancer patients concluded that NLR was a prognostic factor for overall survival, independently of tumour stage. Most patients included in that work and in other publications correspond to early stages of the disease^14^, with only 5^11,13,15–17^ of 15 studies including a smaller proportion (4–14%) of MBC patients. The authors concluded that the prognostic value of NLR was observed both in studies including only early BC and in those with mixed populations of early and metastatic BC, although mixed studies showed statistical heterogeneity. In fact, one of these studies excluded MBC patients from the analysis^17^, and two of them grouped stage III and IV patients for the multivariate analysis^15,16^. Thereby, the main obstacle for a proper interpretation of NLR data in the metastatic setting is the combined analysis with early breast cancer data, including the largest series (n = 197) of MBC in the metanalysis^11^. After the publication of the above-cited metanalysis, one large series of 171 patients^18^ and several other articles including 30–50 patients^8,19,20^, have specifically addressed the value of NLR in MBC patients, with most results showing an association between higher NLR and lower survival or response. More recently, a study focused on good performance status (ECOG 0–1) triple negative metastatic breast cancer (n = 57) treated with chemotherapy has pointed to NLR as a predictive factor for response to platin-based chemotherapy, while no impact of NLR was found in a control group of 148 patients with hormone receptor-positive, HER2-negative MBC. Similarly, NLR was an independent prognostic factor for overall survival (OS) in the TNBC group, but no differences were found in the control group^21^. In many of these works, however, the authors did not include other accepted prognostic variables for MBC, such as type of diagnosis (initial or recurrent MBC) or performance status (ECOG). Thus, although the prognostic value of NLR in early breast cancer seems to be well established, its prognostic meaning in MBC is still uncertain.

Our aim was to determine whether the prognostic value of baseline NLR in an unselected and well-characterized MBC cohort was an independent prognostic or predictive factor or simply a factor dependent on other characteristics of the neoplasm.

## Methods

### Design and eligibility criteria

We conducted a retrospective study of consecutive patients with metastatic breast cancer treated in a single center (Hospital Morales Meseguer, Murcia, Spain) between 2009 and 2016. Patients should have a pathologically confirmed diagnosis of metastatic breast cancer (either as an initial diagnosis or as distant recurrence), with any tumour subtype, performance status or type of treatment, together with availability of pre-treatment differential blood count and information on PFS (first line treatment) and OS. Patients with hematological preexisting conditions or active infection were excluded. Patients were treated according to usual clinical practice criteria.

All procedures performed in this study were in accordance with the ethical standards of the institution and with the 1964 Helsinki declaration and its later amendments. The Institutional Review Board of Hospital Morales Meseguer approved the study (internal code: EST07/15). Design of the study and data presentation follow REMARK recommendations^22^. Informed consent was obtained from all individual participants included in the study.

### Clinical and analytical variables

Clinical data, including performance status, stage at diagnosis, metastases location, type of treatment and pathological data were obtained from electronic medical records. Visceral crisis was defined according to the Advanced Breast Cancer international consensus guidelines^1^. No imputation was performed for missing data. Conventional immunohistochemistry was performed in tumour biopsy (primary or metastasis) to determine estrogen and progesterone receptors, and HER2 was evaluated with immunohistochemistry (Herceptest, DAKO) and FISH according to current practice and international guidelines. We collected the analytical data (absolute lymphocyte and neutrophil count, expressed as n × 10^−6^ L) from laboratory databases, using the pre-treatment differential blood count corresponding to the nearest date to the beginning of first-line treatment. Neutrophil-lymphocyte ratio (NLR) was calculated by dividing the absolute neutrophil count by the absolute lymphocyte count. When available, we obtained the value of NLR at time of early breast cancer diagnosis in those patients with initial M0 stage and metastatic recurrence.

### Statistical analysis

The primary objective of the study was to determine whether pre-treatment NLR values of MBC patients are associated with overall survival, defined as the time between date of diagnosis of metastatic disease and date of death or last follow-up. The secondary objectives were to evaluate the association of NLR with progression free survival (defined as the time between date of first treatment and date of death or disease progression), the subtype of the tumour or other relevant clinical and pathological characteristics; patients without available data on the date of progression were excluded from this analysis.

NLR values are expressed as median and interquartile range (Q1–Q3). We used non-parametric tests (Mann-Whitney U test, Kruskal-Wallis) for the evaluation of differences of NLR between groups. We used Chi-squared test for comparison of proportions between groups. Survival was determined from the date of diagnosis of metastatic disease. Dates of death for overall survival data were confirmed with the national deceased registry (INDEF). We used Kaplan-Meier curves and Cox univariate and multivariate proportional hazard models for OS and PFS analysis. For comparisons, NLR was used both as a continuous and as a discrete covariate, with pre-specification of the median value in the whole group as a cutpoint. Multivariate analysis included all clinical variables considered clinically or statistically significant, without backward or forward stepwise selection.

Based on an expected hazard ratio of 1.60 for overall survival in patients with high (over the median) NLR, with an alpha level of 0.95 and a power of 0.80, we calculated a sample size of 237 patients, with a 10% added for potential information loss, yielding a total sample size of 261 patients. All data were included in a database using SPSS v.21 software (SPSS, Inc, Chicago, IL, USA), and the same software was used for statistical analyses. A p value of <0.05 was set as the limit for statistical significance.

## Results

### Patient characteristics

We included 263 consecutive metastatic breast cancer patients, all of them Caucasian, and 117 of them with metastatic disease at diagnosis (Table 1). Median age was 59 years, and most patients (81.4%) had a good performance status (ECOG 0–1). Visceral disease was present in 60% of patients, and 25 patients (9.5%) showed a visceral crisis, while central nervous system (CNS) involvement at diagnosis appeared in only 7% of patients. Most patients (73%) received first line treatment with chemotherapy, either alone (46%) or in combination with biological agents (bevacizumab or trastuzumab); the rest of patients (27%) received endocrine first line therapy. The most frequent subtype was HER2− HR+ breast cancer, with 24.6% of HER2+ patients and 14.5% with triple negative disease.

**Table 1:** Patient characteristics.

Age	Median (range)	N^a^	%
		59 (19–95)	
Performance status^b^	ECOG 0–1	214	81.4%
	ECOG 2–3	49	18.6%
Histology	Ductal	227	86.3%
	Lobular	27	10.3%
	Other	9	3.4%
Metastases at diagnosis	M0	146	55.5%
	M1	117	44.5%
Estrogen receptor	ER−	60	23.2%
	ER+	199	76.8%
Progesterone receptor	PR−	98	38.1%
	PR+	159	61.9%
HER2	HER2−	193	75.4%
	HER2+	63	24.6%
Tumour subtype	HR+ HER2−	156	60.9%
	HR+ HR+	44	17.2%
	HR+ HR−	19	7.4%
	TNBC	37	14.5%
Metastasis location	Bone/soft part	105	39.9%
	Visceral	65	24.7%
	Visceral + Bone/SP	93	35.4%
Number of metastasis locations	<2 locations	152	57.8%
	>=2 locations	111	42.2%
CNS metastasis	No CNS disease	245	93.2%
	CNS disease	18	6.8%
Visceral crisis	No	238	91.5%
	Yes	25	9.5%
Recurrence during adjuvant treatment	No	64	44.1%
	Yes	81	55.9%
Treatment (1^st^ line)	Endocrine therapy	45	19.8%
	Chemotherapy	115	50.7%
	Chemotherapy and biological agents Anti-HER2 Anti-VEGF	67 48 19	29.5%

Abbreviations: ER: estrogen receptor; PR: progesterone receptor; HR: hormone receptors.

### NLR values and association with clinical and pathological variables

Median NLR for the whole series was 2.32 (range: 0.70–44.33; interquartile interval (Q1–Q3): 1.70–3.50). No correlation with age of patient was found (p = 0.67). As shown in Table 2, the analysis of the distribution of NLR values across the clinical and pathological groups was consistent with a significant association of higher values of NLR with worse performance status (p = 0.008), estrogen receptor negative disease (p = 0.02), visceral metastasis (p = 0.03), visceral crisis (p < 0.001) and CNS metastasis (p = 0.006).

**Table 2:** NLR values across MBC patient subgroups.

N = 263		NLR		P^*^
		Median	Interquartile interval (Q1–Q3)	
Performance status	ECOG 0–1	2.16	1.59–3.40	**0.008**
	ECOG 2–3	2.93	2.07–3.79	
Histology	Ductal	2.32	1.68–3.50	0.86
	Lobular	2.13	1.70–3.42	
Metastases at diagnosis	M0	2.15	1.57–3.40	0.08
	M1	2.50	1.81–3.71	
Estrogen receptor^a^	ER negative	2.75	2.03–4.08	**0.02**
	ER positive	2.16	1.61–3.33	
Progesterone receptor	PR negative	2.41	1.71–3.50	0.70
	PR positive	2.29	1.71–3.62	
HER2	HER2 negative	2.23	1.65–3.40	
	HER2 positive	2.58	1.81–4.00	
Tumour subtype	HR+ HER2−	2.13	1.60–3.15	0.09
	HR+ HR+	2.35	1.75–3.51	
	HR+ HR-	3.23	2.16–4.17	
	TNBC	2.41	1.92–4.00	
Metastasis location	Bone/soft parts (SP)	2.13	1.59–3.00	**0.04**
	Visceral	2.36	1.57–3.58	
	Visceral + Bone/SP	2.46	1.83–5.10	
Number of metastasis locations	<2 locations	2.16	1.59–3.30	0.06
	> = 2 locations	2.50	1.81–4.25	
Visceral disease	Yes	2.44	1.75–4.18	**0.03**
	No	2.13	1.59–3.00	
CNS metastasis	No CNS disease	2.25	1.65–3.42	**0.006**
	CNS disease	3.14	2.50–6.21	
Visceral crisis	No	2.24	1.64–3.33	**<0.001**
	Yes	4.83	2.08–8.86	
Recurrence during adjuvant treatment	No	2.24	1.64–3.70	0.45
	Yes	2.12	1.55–3.00	

*Mann-Whitney U-text or Kruskal-Wallis test.

The evolution of NLR between early breast cancer diagnosis and metastatic recurrence was analysed in a subset of patients with available laboratory data (n = 93), showing higher values of NLR for metastatic disease (median NLR of 2.22, Q1–Q3 1.47–3.40, vs. 1.65, Q1–Q3 1.25–2.24 for early disease; p < 0.001, Wilcoxon signed rank test). NLR increased in 67.7% of patients from time of diagnosis of early disease to time of metastatic recurrence.

### Progression free and overall survival association with NLR

At time of last analysis (30-May-2018), with a median follow-up of 44.9 months (range, 6–107), 170 patients have died, with a median overall survival of 36.2 months (95%CI 29.9–42.6). Progression free survival (n = 227) to any first-line treatment was 12.0 months (95%CI 10.7–13.3), with 44 patients without events.

**Figure 1:**
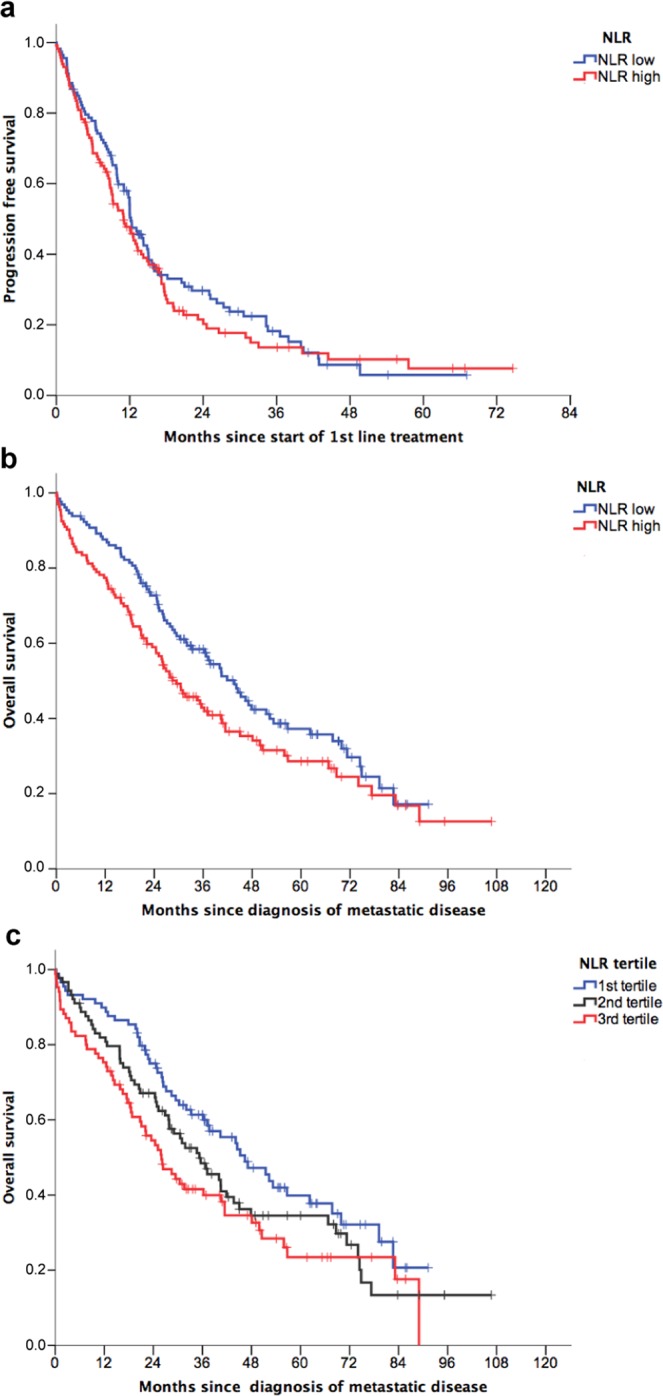
Survival analysis of metastatic breast cancer patients according to baseline NLR. (**a**) Kaplan-Meier curves for progression free survival (N = 227) according to dichotomized NLR (classified as low or high using the median value as cut-point of 2.32); log-rank test, P = 0.43. (**b**) Kaplan-Meier curves for overall survival (N = 263) according to dichotomized NLR; log-rank test, P = 0.048 (**c**) Kaplan-Meier curves for overall survival (N = 263) according to NLR tertiles.

Univariate analysis of NLR as a continuous variable for PFS showed an association of higher NLR with a higher risk of progression after 1^st^ line treatment (Table 3). No differences were found for PFS using median value as a cut-off (p = 0.43) (Fig. 1a). In the multivariate analysis, which included all covariates significant in the univariate analysis, NLR was not an independent factor for PFS (p = 0.21). Results did not change after stratification by type of treatment (chemotherapy versus other).

**Table 3:** Univariate and multivariate analysis for progression free survival (n = 227).

	Univariate			Multivariate		
	Beta	HR (95%CI)	P*	Beta	HR (95%CI)	P**
M1 at diagnosis	−0.475	0.62 (0.46–0.84)	0.002	−0.384	0.68 (0.49–0.94)	**0.02**
ER negative	0.691	1.99 (1.43–2.78)	<0.001	0.360	1.43 (0.96–2.15)	0.08
PR positive	−0.590	0.55 (0.41–0.75)	<0.001	−0.340	0.71 (0.50–1.02)	0.06
HER2+	0.065	1.07 (0.76–1.50)	0.71	—	—	—
ECOG 0–1	−0.487	0.61 (0.43–0.88)	0.008	−0.426	0.65 (0.43–0.98)	**0.04**
NLR (continuous)	0.044	1.045 (1.005–1.086)	0.03	0.028	1.029 (0.984–1.075)	0.21
NLR high (>2.32)	0.117	1.12 (0.84–1.50)	0.43	—	—	—
>=2 locations	−0.372	0.69 (0.51–0.92)	0.01	—	—	—
CNS metastasis	−0.258	0.77 (0. 42–1.42)	0.41	—	—	—
Visceral metastasis	0.514	1.67 (1.24–2.26)	0.001	0.291	1.34 (0.97–1.84)	0.08
Visceral crisis	1.003	2.73 (1.70–4.37)	<0.001	0.505	1.66 (0.97–2.84)	0.07

**Figure 2:**
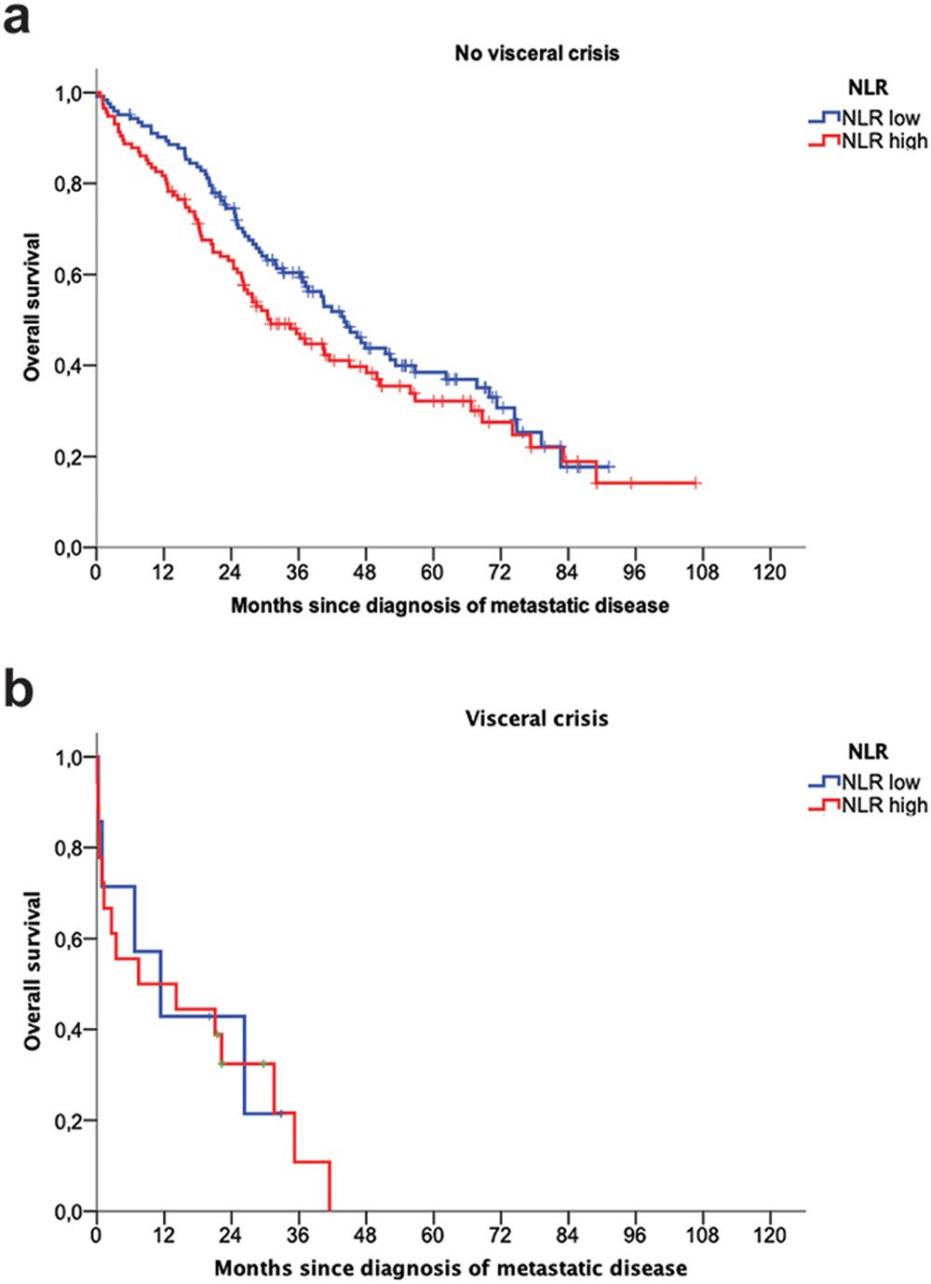
Overall survival Kaplan-Meier curves of metastatic breast cancer patients with and without visceral crisis according to baseline NLR. (**a**) Kaplan-Meier curve for overall survival in patients without visceral crisis (N = 238) according to dichotomized NLR (classified as low or high using the median value as cut-point of 2.32); log-rank test, P = 0.14 (**b**), Kaplan-Meier curve for overall survival in patients with visceral crisis (N = 25) according to dichotomized NLR; log-rank test, P = 0.94.

The same analysis was performed for overall survival (n = 263). In the univariate analysis, high NLR (using the median value as cut-off) was associated with a significantly worse overall survival (Fig. 1b) and this association followed a dose-response pattern as shown in the Kaplan-Meier curves for the NLR tertiles (Fig. 1c). As shown in Table 4, in the univariate analysis, NLR showed a significant impact on overall survival both as a continuous variable (HR 1.06; 95%CI 1.02–1.10) and as a discrete variable (HR 1.36, 95%CI 1.00–1.83). In the multivariate analysis, the final model included metastasis at diagnosis, ER, performance status (ECOG), CNS involvement, visceral disease and visceral crisis as independent covariates, but NLR no longer showed a prognostic impact (p = 0.29). Stratification by visceral disease and exclusion of CNS metastasis did not change the general model (data not shown). Similarly, as shown in Fig. 2, NLR was not predictive of patients’ outcome when MBC patients were stratified according to visceral crisis (p = 0.14 and p = 0.94 for patients without and with visceral crisis, respectively; log-rank test).

**Table 4:** Univariate and multivariate analysis for overall survival (n = 263).

	Univariate			Multivariate		
	Beta	HR (95%CI)	P*	Beta	HR (95%CI)	P**
Age	0.011	1.012 (1.001–1.022)	0.028	0.009	1.01 (0.99–1.02)	0.12
M1 at diagnosis	−0.351	0.70 (0.52–0.96)	0.03	−0.548	0.58 (0.41–0.81)	**0.002**
ER negative	0.985	2.68 (1.91–3.76)	<0.001	0.637	1.89 (1.22–2.93)	**0.005**
PR positive	−0.650	0.52 (0.38–0.71)	<0.001	−0.329	0.72 (0.48–1.07)	0.10
HER2 +	0.008	1.01 (0.70–1.44)	0.97	—	—	—
ECOG 0–1	−1.038	0.35 (0.25–0.50)	<0.001	−1.121	0.32 (0.22–0.48)	**<0.001**
NLR (continuous)	0.058	1.06 (1.02–1.10)	0.002	0.025	1.02 (0.98–1.07)	0.29
NLR high (>2.32)	0.305	1.36 (1.00–1.83)	0.048	0.110	1.12 (0.80–1.56)	0.52
> = 2 locations	0.629	1.87 (1.38–2.54)	<0.001	—	—	—
CNS metastasis	1.147	3.15 (1.93–5.15)	<0.001	0.968	2.63 (1.51–4.57)	**0.001**
Visceral metastasis	0.689	1.99 (1.44–2.75)	<0.001	0.342	1.41 (0.98–2.01)	0.06
Visceral crisis	1.206	3.34 (2.07–5.39)	<0.001	0.884	2.32 (1.31–4.11)	**0.004**
**Composite model**						
Prognostic score	0.395	1.48 (1.38–1–60)	<0.001	0.389	1.47 (1.37–1.59)	**<0.001**
NLR (continuous)	0.044	1.045 (1.005–1.086)	0.03	0.024	1.02 (0.98–1.07)	0.28
NLR high (>2.32)	0.117	1.12 (0.84–1.50)	0.43	0.136	1.15 (0.84–1.57)	0.39

In order to better define the prognostic value of NLR when compared with the rest of variables, we generated a single composite score obtained as the summation of the weighted scores for each covariate of the multivariate model (ECOG > 2: 3 points; M0 at diagnosis, ER negative, CNS metastasis and visceral crisis, 2 points each; visceral metastasis: 1 point). The prognostic score, ranging from 0 to 10 in our cohort, showed a good performance, with a HR of 1.48 (95%CI, 1.38–1.60) and estimated overall survivals from 71.3 months (95%CI, 63.8–78.8) for the group of patients with 0–1 points to 2.6 months (95%CI, 0.28–4.9) for those with the highest score (>7). When NLR was included as a covariate together with the single prognostic score in a Cox model, we found an independent significant impact neither as a continuous variable nor as a dichotomized variable (Table 4).

## Discussion

NLR has shown in multiple works and at least two metanalysis^14,23^ its prognostic relevance in early breast cancer, with higher values associated to worse overall and disease free survival. However, only discordant and mixed results are available in the metastatic setting. In this work, we have evaluated the prognostic and predictive meaning of baseline NLR in a cohort of 263 unselected MBC patients. Taken together, our data show that NLR is an independent predictor neither of OS nor of PFS, suggesting that its prognostic value, which is apparently clear in the univariate analysis, depends on other covariates, such as metastasis location, performance status and stage at diagnosis.

Our results contrast with other published results. At least part of this difference might be related to the inclusion of performance status as a prognostic covariate in our series: a worse ECOG was significantly associated to higher NLR in our patients, and the absence of this variable in other studies^11,18–20^ may increase the apparent contribution of NLR to prognosis. A second prognostic factor that was considered in our work was the stage IV at diagnosis: in this case, no significant differences in NLR were observed between patients with MBC at diagnosis and those presenting metastasis as a result of distant recurrence, but, similarly to other publications, stage M1 was associated with longer overall survival. Third, CNS involvement, which has been associated to higher NLR in other settings, is usually not included in other NLR studies with BC patients. In our work, CNS involvement was a strong prognostic factor, thus included in the final model for OS; however, the elimination of CNS disease did not change the model and consequently this factor is not apparently the main cause of NLR loss of statistical significance. Similarly, visceral crisis seemed to carry the main prognostic weight of visceral disease and was associated with a higher NLR, but the stratification by this factor did not show a prognostic impact of NLR among metastatic breast cancer patients without visceral crisis. Finally, an overall survival model in which a composite prognostic score was included as the only covariate together with NLR again resulted in the lack of significance of NLR. Since the key question for considering a biomarker as clinically useful is whether it adds new information to the established clinical prognostic or predictive factors, our results do not support the introduction of NLR as a useful prognostic or predictive factor in the overall population of MBC patients. The results observed in other series in which small samples of MBC patients are analysed together with larger samples of early breast cancer patients seems to be mainly justified by the prognostic value of NLR in early disease, and by the absence of some covariates, only relevant to MBC, in the multivariate analysis. Thereby, NLR seems to have a greater prognostic impact in the early disease setting, according to the considerable amount of data included in the most recent metanalysis^14^ and to the additional evidence provided by works controlling for other prognostic factors^12^.

A second question is whether NLR might be prognostic and potentially useful in a particular MBC subgroup. One work has pointed to bone metastasis as a particular situation in which NLR might be useful as a prognostic biomarker^24^. In our patients, stratification by visceral versus non-visceral disease did not change the prognostic impact of NLR. Since the work by Wang *et al*. included multiple primary tumours (lung, gynaecological and digestive) and the MBC patients are not analysed separately, their results might be well justified by the inclusion of non-MBC patients^24^. Race is another potential confounding factor, since many of the MBC patients included in the metanalysis^11,14^ and virtually all MBC patients included in more recent studies^18–20^ are of Asian ancestry. In a previous metanalysis^23^, a lower HR and a higher heterogeneity was observed in Western studies addressing the association between NLR and BC prognosis, and the influence of race might be also relevant in the metastatic setting.

However, perhaps the main question is the relative importance of NLR in the different BC subtypes: in the early BC setting, one metanalysis showed that the prognostic value of NLR was clear for HER2+ and TNBC disease, while no differences were apparent in luminal A and B breast cancer^23^. A second and larger metanalysis^14^ including both early and metastatic BC series, showed a greater effect of NLR on DFS for ER-negative and HER2-negative patients, while the effect of NRL on OS did not depend on the tumour subgroup. Other works have shown conflicting results, with a higher impact of NLR in luminal early BC^25^. However, as previously stated, these results are driven by the early BC cases and their relevance for MBC is uncertain. In the metastatic setting, another recent work focused on TNBC, showing that a high NLR is predictive of both a worse OS and PFS after treatment with platin, although the TNBC group and the HR+ HER2− control group included patients in 2^nd^ or latter lines of treatment^21^. We did not reproduce these results in the first line setting, even after stratifying the model by tumour subtypes or estrogen receptor status, although this analysis was limited by the sample size (n = 37, TNBC; n = 63, HER2+).

Our work has several limitations. First, the sample size is small, and, while we can reject the hypothesis of a meaningful (HR > 1.60) prognostic contribution of NLR, we cannot totally discard a minor prognostic effect of NLR in MBC, although in such case its importance would be less relevant. The sample size has also limited the analysis of NLR across major biological subtypes of breast cancer. Finally, first line treatment was heterogeneous, according to the variable clinical characteristics and tumour subtype of our patients. We did not find differences after stratification by chemotherapy versus no chemotherapy, but the predictive value of NLR might be different in well-defined and homogeneous treatment settings, such as MBC patients treated with immunotherapy or after complete response to chemotherapy^18^. However, the main strength of our data is the availability and inclusion in the model of other prognostic variables such as performance status, not included in most previous publications^11,18–20^, stage at diagnosis, visceral crisis and CNS involvement, all of them recognized as relevant prognostic clinical variables in MBC. These covariates seem to be a likely explanation for a large part of the prognostic variability attributed to NLR.

In conclusion, our results show that, although NLR is increased with metastatic recurrence and a higher NLR is present in MBC Caucasian patients with worse prognosis, NLR is not an independent factor for overall survival or progression free survival in MBC when other factors, especially performance status, visceral crisis and stage at diagnosis (M0 vs. M1), are considered. These data are in contrast with those found in early breast cancer. Population-based analysis or pooled analysis of existing series might be useful to further clarify whether NRL might contribute to prognostic stratification in some subgroups of metastatic breast cancer patients. In addition, the exploration of NLR might be also valuable in the growing group of MBC patients treated with immunotherapy.

## Data Availability

The datasets analysed during the current study are available from the corresponding author on reasonable request.
